# What's new in emergencies, trauma, and shock? Snake envenomation and organophosphate poisoning in the emergency department

**DOI:** 10.4103/0974-2700.43180

**Published:** 2008

**Authors:** Praveen Aggarwal, Nayer Jamshed

**Affiliations:** Division of Emergency Medicine, All India Institute of Medical Sciences, New Delhi, India. Email: peekay_124@hotmail.com

## SNAKE ENVENOMATION

Snakebite is a major problem worldwide. According to estimates, more than 5 million venomous snakebites occur every year, and nearly 125,000 of those who suffer snakebite die. The majority of the deaths occur in the rural population because of inadequate primary treatment and lack of tertiary care facilities. However, there are now reasons to be optimistic as better management and advances in the treatment of snakebite can bring down the morbidity and mortality drastically.

Prehospital management of snakebite victims is important. The most important aspect of prehospital care is to reassure the patient and immobilize the bitten extremity. Incision and suction and use of electric shock therapy are not recommended. For neurotoxic snakebites, a bandage may be wrapped snugly around the entire length of the bitten extremity so as to reduce dissemination of venom by slowing lymph flow.[1] Since a bandage is not recommended for viper bites, this recommendation may not be of much value as most victims fail to recognize the snake bitting them.[2]

In the hospital, administration of anti-snake venom (ASV) is the mainstay of treatment. The correct dose and timely administration of ASV and supportive therapy may affect the outcome and rate of complications.[2,3] The most commonly used ASV, particularly in developing countries, is the equine-derived one, which is polyvalent and carries the risk of anaphylaxis. A skin test is presently not recommended before administering equine ASV.

There is no consensus on the dose of ASV required in the management of snakebite. The initial dose varies from 2 vials to 20 vials, but most physicians agree that the initial dose should be around 8–10 vials so that the ASV could bind most of circulating venom before it gets fixed to the tissues. Similarly, the timing and quantity of repeat doses can also vary. Presently, it is recommended that in hemotoxic envenomation, a repeat dose should be administered after 6 h if coagulation is not restored (i.e., bleeding continues or whole blood clotting time remains above 20 min). This is because the liver normally takes about 6 h to restore clotting factors. In case of neurotoxic snakebite, a repeat dose needs to be administered after 1–2 h if the patient does not improve.[2,4] After that period, tissue-bound venom cannot be neutralized with additional doses of ASV. This strategy is of particular importance in developing countries where ASV is often in short supply. Another area of recent interest is the use of lower doses of ASV which may be as effective as high-dose treatment in controlling the features of envenomation. This will greatly reduce the cost of treatment.[5]

Since equine ASV carries the risk of severe reactions, Crotalidae polyvalent immune Fab antivenom (FabAV) produced from sheep, has been is use in the USA since the year 2000. It is about 5.2 times more potent than equine-derived ASV, though its half-life is shorter. It is remarkably stable under extreme conditions and has almost no risk of anaphylaxis. The recommended initial dose is 4–6 vials. If local manifestations are not controlled and the coagulation profile does not return to normal, an additional 4–6 vials should be repeated every hour until initial control is achieved. After initial control is achieved, it is recommended to give an additional 2 vials every 6 h for up to three doses.[6] Repeated doses are required due to the short half-life of FabAV. In India, anti-viper venom and antivenom against king cobra and krait are being developed from egg yolk of Leghorn chicken.[7] This will be an important development as equine ASV is costly and associated with several adverse effects.

Snake venoms are chemically complex mixtures of proteins, many of which have enzymatic properties. Local tissue damage (edema and necrosis) and some systemic effects like hemorrhage are produced by the enzymes, metalloproteinases. Several inhibitors of metalloproteinases are being studied to reduce the local effects of snake venom. Metalloproteinase inhibitors like batimastat and neo-clerodane diterpenoid have been shown to decrease systemic toxicity as well as local hemorrhage, edema, and dermal necrosis following administration of snake venom in experimental animals.[8] Synthetic metalloproteinase inhibitors may become useful therapeutic tools in the future for treating patients with snakebite.

Another area of interest is targeting VEGFR (vascular endothelial growth factor receptors) by using VEGFR inhibitors. Some toxins in snake venoms act like VEGF and cause increased capillary permeability through stimulation of VEGFR-1 and VEGFR-2.[9]

## ORGANOPHOSPHATE POISONING

Organophosphate (OP) poisoning is a major global health problem with more than 200,000 deaths every year.

OP poisoning leads to three main syndromes: acute cholinergic syndrome, intermediate syndrome (IMS), and OP-induced delayed polyneuropathy (OPIDPN). Acute cholinergic syndrome occurs due to inhibition of acetylcholinesterase (AChE), resulting in excessive accumulation of acetylcholine (ACh) at the pre- and post-ganglionic parasympathetic and sympathetic nervous system, central nervous system, and skeletal muscle motor endplates. Excess of ACh stimulates muscarinic and nicotinic receptors resulting in acute cholinergic features, including excessive pulmonary secretions, muscle weakness, and CNS depression. Binding of OP with AChE is initially reversible, but after the passage of time this binding becomes irreversible (due to aging of AChE). IMS occurs 24–96 h after exposure and presents with weakness of neck flexors, proximal weakness, cranial nerves involvement, and respiratory muscle paralysis. Mortality is generally related to acute cholinergic syndrome and IMS.

It is difficult to predict which patient will develop IMS following OP exposure. Recently, repetitive nerve stimulation (RNS) done sequentially has been studied in patients with acute OP poisoning. Characteristic changes in RNS occurred preceding the development of IMS. These changes included a decrement–increment pattern during the early stage of intoxication, which worsened to a severe decrement pattern at high-frequency stimulations before development of IMS.[10] Thus, serial electrophysiological studies could be used for objective clinical assessment in the management of patients following OP poisoning and in research (such as evaluation of the effects of therapeutic agents or a therapeutic regimen).

The currently available treatment of OP poisoning is mainly based on combined administration of an AChE reactivator [pralidoxime (PAM) or other oximes], a muscarinic receptor antagonist (atropine), and an anticonvulsant (diazepam), along with supportive care.

The position statement by the American Academy of Clinical Toxicology and European Association of Poison Centers and Clinical Toxicologists states that gastric lavage should be considered only if a poisoned patient presents within 1 h of ingestion of a potentially toxic amount of a poison. However, gastric lavage is routinely used, particularly in developing countries, where the incidence of OP poisoning is quite high. This is due to lack of any good studies conducted on the role of gastric lavage in OP poisoning. Hopefully, the results of a large study being conducted in China will settle this issue. Till then, it may be prudent to perform a lavage in patients who present soon after ingestion of a substantial amount of OP.

The utility of multiple doses of activated charcoal has been studied in OP poisoning and it has been shown that multiple doses do not carry additional benefits compared to a single dose of activated charcoal.[11,12]

Atropine is used to counter the muscarinic effects of OP, particularly the cardiovascular effects and effects on bronchial secretions. As many as 30 dose schedules of atropine have been cited by different authors. Since the requirement of atropine to counter muscarinic effects may be very high, repeating the same dose every 3–10 min will at times take several hours to produce atropinization. The aim of atropine therapy is to improve cardiac and respiratory function as quickly as possible. It is therefore recommended that the initial dose of atropine be doubled every 5 min till clear-cut endpoints are achieved. These endpoints include pulse > 80/min, systolic blood pressure > 80 mmHg, and rapid reversal of bronchospasm and bronchorrhea. Once the endpoints are reached, atropine is given as an infusion, at the rate of 10–20% of the total initial dose every hour.[13] Since atropine may produce serious central nervous system toxicity, the use of glycopyrrolate which does not cross the blood-brain barrier has been studied.[14] However, currently there is no evidence to suggest better efficacy of glycopyrrolate as compared to atropine.

Oximes have been widely used in patients with OP poisoning. However, a meta-analysis of various trials did not conclusively prove the efficacy of PAM.[15] Unfortunately, in the trials that were analyzed, PAM was used in doses lower than that recommended by WHO. A recent trial reported from India showed beneficial effects with high doses of PAM in patients with moderately severe OP poisoning, particularly when it is given within 2.5 h of exposure.[16] The initial dose was 2 g of PAM (as the iodide salt) followed by 1 g every hour as an infusion for 48 h, and then 1 g every 4 h until recovery. It is important to note than pralidoxime chloride, which is used in most countries, is about 1·5 times more potent than the iodide salt. Further, oximes may only reverse the inhibition of AChE produced by diethyl OPs (e.g., parathion and quinalphos), while having little effect on AChE inhibited by dimethyl OPs (e.g., monocrotophos and oxydemeton-methyl). This may be related to early aging of AChE with dimethyl OPs as compared to diethyl OPs. Use of high-dose PAM may however be effective in both diethyl- and dimethyl-OPs.[16]

Since very few patients present within the first few hours of ingestion, when oximes are more likely to be beneficial, several other therapies have been tried in experimental as well as in human studies.

Studies have shown that overstimulation of the central receptors by OP compounds may contribute to early death. In addition, these compounds may reduce phrenic nerve output due to action on the respiratory center. Diazepam has been used to control seizures in OP poisoning; it now appears that diazepam may also be beneficial in patients without seizures, in whom it may protect the CNS and reduce mortality.[17,18]

Bioscavengers have been used as prophylactic agents in military personnel to reduce the effects of possible nerve agent exposure. These include cholinesterases (e.g., butyrylcholinesterase or BChE) that bind and neutralize OP.[19] Equine BChE produces anti-enzyme antibodies and is therefore not suitable for human use. Purified human BChE has been tested successfully in animal models and may be useful in humans at high risk of nerve agent poisoning.[19–21] However, such a prophylactic approach is not practical for self-poisoning with OP.

An alternative to bioscavenger therapy is the use of human plasma, which is rich in cholinesterase. In a small study of 33 patients with OP poisoning, use of plasma in addition to atropine and PAM was found to reduce mortality and occurrence of IMS.[22] Another approach is to use recombinant bacterial phosphotriesterases or hydrolases. These enzymes break down OP pesticides and may confer protection.[23,24] Trials are needed to confirm their efficacy in human cases of OP poisoning.

Accumulation of ACh in the synaptic cleft is generally considered as the main cause of the symptoms that ultimately lead to death. A drug which decreases ACh release in the brain and muscle can prevent and counteract the convulsions that occur in OP poisoning and may improve survival rate. It has been found that adenosine A1-receptor agonists reduce ACh release in the brain and in muscles.[25] Compared to oxime treatment, administration of A1 adenosine agonists may provide protection against all types of OPs, including nerve agents. These agents are likely to be effective even after aging of AChE. Cardiovascular effects are the main adverse reactions to A1 adenosine agonists. Important categories of drugs being investigated include N6-cyclopentyladenosine (CPA), 5′-N-ethylcarboxamido-adenosine (NECA), and deoxyribose analogues of N6-cyclopentyladenosine (CPA).[25,26]

Magnesium reduces ACh release from presynaptic terminals by blocking ligand-gated calcium channels in cardiac muscle. This may reduce OP-induced QT interval prolongation and ventricular tachycardia. Beneficial effects of magnesium have been shown in a small study where magnesium sulphate was administered in a dose of 4 g over 24 h.[27]

Sodium bicarbonate has been shown to improve outcome in animal models of OP poisoning.[28] Postulated mechanisms for beneficial effects of sodium bicarbonate include enhanced inactivation of OP by hydrolysis, improved tissue perfusion through volume expansion, improved efficacy of oximes, and a direct beneficial effect on the neuromuscular junction. However, insufficient evidence exists at present to recommend sodium bicarbonate as a therapeutic option in humans poisoned with an OP.[29]

Charcoal hemoperfusion may be effective in poisoning with some OP compounds. It has been shown to be effective in patients of dichlorvos poisoning.[30] Dichlorvos has poor fat solubility and low volume of distribution.

Since OP poisoning carries a high mortality, poor prognostic factors need to be assessed in the emergency department. Use of the modified APACHE II score which is much simpler and less time-consuming compared to APACHE II score has been shown to be effective for prognostication in the OP-poisoned patients in an emergency situation.[31] Similarly, the Glasgow Coma Score (GCS) was found to be a good indicator of prognosis, particularly in patients with dimethoate poisoning (the predictive value was less accurate for fenthion poisoning).[32] Using GCS ≤ 13 as the cutoff, patients could be divided into a high-risk group with 37% case fatality rate and a low-risk group with only 4% mortality. The outcome based on GCS was similar to that based on a more complicated Poison Severity Score developed by the International Program on Chemical Safety. Thus, this study suggests that in emergency departments patients of OP poisoning with GCS ≤ 13 are at higher risk of death and should, therefore, receive intensive monitoring.

## CONCLUSION

Emergency physicians need to optimistic about the battle against snakebite. Future research in snakebite treatment will rely largely on the identification of species-specific venom and use of ovine Fab and egg yolk Fab anti-snake venoms. Medical management of organophosphate pesticide poisoning requires optimal doses of atropine and oximes along with good supportive care. The role of newer agents in OP poisoning remains experimental at present but it is hoped that human trials in the next few years will help in formulating a comprehensive management protocol in patients with OP poisoning, and will reduce the high mortality associated with it.
